# Principal nonlinear dynamical modes of climate variability

**DOI:** 10.1038/srep15510

**Published:** 2015-10-22

**Authors:** Dmitry Mukhin, Andrey Gavrilov, Alexander Feigin, Evgeny Loskutov, Juergen Kurths

**Affiliations:** 1 Institute of Applied Physics of the Russian Academy of Sciences, 603950 Nizhny Novgorod, Russia; 2 Potsdam Institute for Climate Impact Research, 14412 Potsdam, Germany

## Abstract

We suggest a new nonlinear expansion of space-distributed observational time series. The expansion allows constructing principal nonlinear manifolds holding essential part of observed variability. It yields low-dimensional hidden time series interpreted as internal modes driving observed multivariate dynamics as well as their mapping to a geographic grid. Bayesian optimality is used for selecting relevant structure of nonlinear transformation, including both the number of principal modes and degree of nonlinearity. Furthermore, the optimal characteristic time scale of the reconstructed modes is also found. The technique is applied to monthly sea surface temperature (SST) time series having a duration of 33 years and covering the globe. Three dominant nonlinear modes were extracted from the time series: the first efficiently separates the annual cycle, the second is responsible for ENSO variability, and combinations of the second and the third modes explain substantial parts of Pacific and Atlantic dynamics. A relation of the obtained modes to decadal natural climate variability including current hiatus in global warming is exhibited and discussed.

Natural variability plays a key role in climate response to different external forcings including carbon dioxide emission. There are several strong internal modes of the climate system, such as El-Niño Southern Oscillation (ENSO)^1^, Pacific decadal oscillation (PDO)^2^, Atlantic Meridional oscillation^3^, which contribute to both regional and global climate, and have a strong impact on global mean temperatures. Recent papers showed that decadal variability of these modes modulates substantially the anthropogenic growth of global mean temperatures^4^,^5^,^6^; in particular, current hiatus in global warming^6^,^7^ is closely connected to the negative PDO phase^6^,^8^,^9^ that cooled the Pacific ocean and shifted ENSO variability toward domination of La-Niña conditions. Currently it is well understood that the ability to predict the behavior of such natural modes is crucial for correct global climate projections; however, reflecting the decadal variability by climate models is not confidence enough^6^,^10^.

In this work an alternative, empirical way to analyze natural modes of climate is proposed: we attempt to resolve empirically dominant hidden signals which actually govern the observed behavior, so as they could be suitable for a low-dimensional dynamical description of climate variability on the considered time scales. In fact, our aim is to reduce the data dimension by means of expanding measured spatio-temporal observations into a few number of modes, which would form a basis for a dynamical system’s phase space construction capturing main features of the observed dynamics.

There is a set of widely used approaches for obtaining principal components by various linear rotations of multivariate data in space domain (see overview of various methods^11^,^12^); they include both traditional empirical orthogonal function (EOF) decomposition and more advanced rotations, which were successfully applied in climate science: in particular, empirical orthogonal teleconnections^13^ and varimax rotation^14^. Also, there are several spatio-temporal extensions of those techniques, based on multichannel singular spectral analysis (MSSA)^15^,^16^,^17^ taking into account time-lag correlations in data. In recent papers advances of MSSA-based expansion for empirical forecast of complex system^18^,^19^ as well as for studying synchronization and clustering in multivariate dynamics^20^ were demonstrated. Papers^21^,^22^ suggest a method for principal mode extraction combining data rotation and system’s evolution operator construction.

A quite apparent inherent drawback of such rotations is regarded to their linearity: many so obtained components contain fractions of the same time scales, or, in other words, modes corresponding to different time scales are usually mixed in different components. Indeed, this effect is essential when there is an evidence of strong nonlinear couplings between time series at different grid points; see the model example in Supplementary Methods. Here we suggest a way for revealing such nonlinear couplings and constructing nonlinear transformation of data to new variables – the nonlinear dynamical modes (NDMs), that is hidden time series holding an essential part of the dynamics. The suggested approach is based on a direct analysis of the data without any prior knowledge about first-principal models. This is in fact a nonlinear generalization of EOF decomposition: instead of constructing linear constraints in the data space, we try to resolve NDMs as most principal nonlinear manifolds (curves) and project data on them. Actually, it is a sort of construction of principal curves^23^ allowing an expansion of vector time series 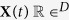 (e.g. discretized climatic spatial field) into the set of dominant parametrically defined curves


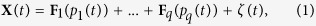


each of which is a map of some hidden (latent) value of 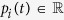 into the data space 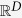. Note that the used method allows reconstructing both latent time series *p*_*i*_(*t*) interpreted as internal modes driving observed variability, and maps **F**_*i*_ needed for analysis of NDMs represented on the data (geographic) grid (hereinafter we will use the abbreviation NDM for both time series *p*_*i*_(*t*) and its image **F**_*i*_(*p*_*i*_(*t*)) in data space). This is in contrast to isomap methods based on principal component analysis (PCA) kernels^24^, which recently found application to the analysis of climate data^25^,^26^: they permit only obtaining principal nonlinear components without an inverse transformation.

A geometrical interpretation of a NDM is also useful: generally, each function **F**_*i*_(.) in (1) defines a nonlinear curve in 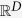. Then the points of the corresponding time series *p*_*i*_(*t*_1_), ..., *p*_*i*_(*t*_*N*_) are just coordinates of *N* points along such a curve. These points are specific projections of the observed data (see Supplementary Figure S5 for illustration).

We developed a Bayesian procedure for a reconstruction of principal modes defined by (1); see details in the section Methods and in Supplementary Materials. Note that the proposed method does allow us to fit some predefined functions **F**_*i*_(.) as well as to find values of hidden time series *p*. Also, it includes very important step, which prevents over-fitting: selection of optimal complexity of decomposition (1), from a statistical point of view. This step is based on Occam Razor principal stating that minimal but explaining things model is the best one: it answers the question of how many nonlinear modes can be resolved from available data. Moreover, it yields the optimal nonlinearity degree of the functions **F**_*i*_(.) as well as prior correlation properties of *p*-time series, which control smoothness of the obtained principal curves in data space. In practice, such prior gives us an efficient separation of time scales between NDMs; hence, different NDMs turn out to be responsible for different modes of natural variability. As a result, we obtain a set of statistically significant nonlinear structures **F**_*i*_(.) and “noise” *ζ*(*t*) – the time series of the residual field. In the next section results of our nonlinear expansion of SST time series is demonstrated and analyzed.

## Results

### Observed time series and data expansion

For analysis we took the time series of NOAA OI.v2 SST monthly field^27^,^28^. This space-distributed time series covers the globe with a resolution of 1° × 1° and has a duration of a bit more than 33 years – from Nov. 1981 to present. Altogether we have 44,219 SST signals in different grid points and our aim is to describe them by a moderate number of principal nonlinear dynamical modes (NDMs) *p* – the set of scalar time series capturing the more the better part of observed variability. To this end, we applied our expansion defined through (1) to this data using the methodology expounded briefly in Methods and in more detail in Supplementary Materials.

**Figure 1:**
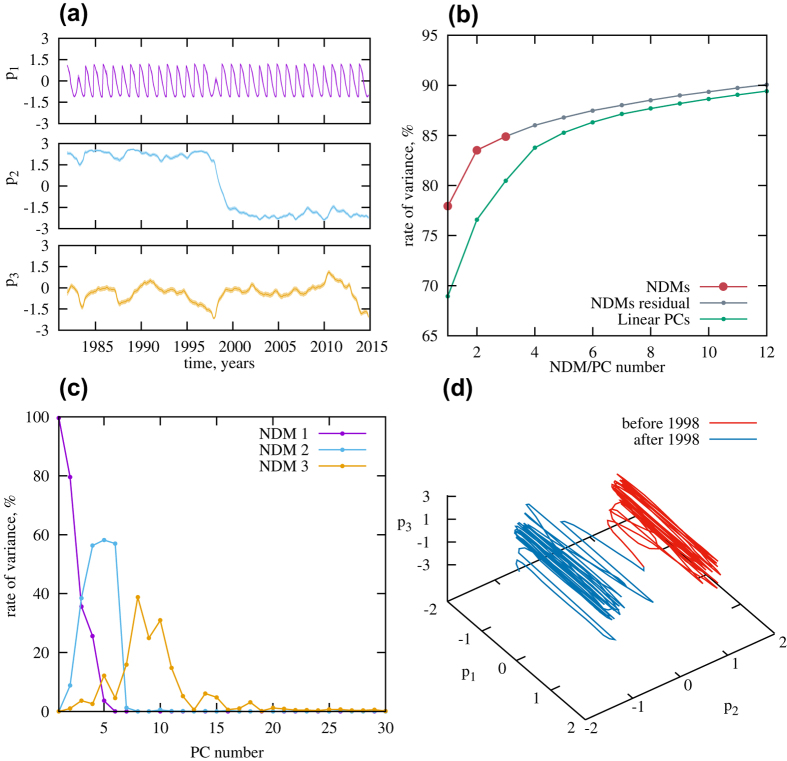
Nonlinear dynamical modes (NDMs) reconstructed from data. (**a**) Hidden time series *p*(*t*) of NDMs placed in order of descending captured variances. (**b**) Rate of cumulative variances captured by modes. Big red points correspond to NDMs, gray dots – to principal components (PCs) obtained from linear EOF decomposition of residuals. Green dots mark the rate of the variances captured by linear PCs of the data. (**c**) Contributions of NDMs to different linear PCs (rates of variance are shown). (**d**) Projection of system’s phase space formed by the reconstructed variables *p*_1_, *p*_2_, *p*_3_. Blue color corresponds to the epoch before 1998, red color – after 1998. Gnuplot (Copyright 1986–1993, 1998, 2004 Thomas Williams, Colin Kelley) was used to create the graphs in this figure.

We applied our decomposition and found that three principal nonlinear modes can be identified; the corresponding three time series *p*(*t*) are shown in Fig. 1a in order of descending (from top to bottom) energy, i.e. captured variance of SST. The line thickness on this figure reflects the 95% Bayesian confidence interval characterizing the uncertainty of the hidden state *p*(*t*) estimation (see Supplementary Methods for details). Further expansion of the rest of data, which is not explained by these three nonlinear modes, comes down to traditional EOF decomposition, simply revealing linear relationships between time series. Comparing the amount of total variance of data captured by NDMs vs. traditional linear principal components (PCs) obtained by projecting data to the leading EOFs, we find that the three NDMs explain 85% of whole data variance – the same amount as from approximately 6 linear PCs; see Fig. 1b displaying cumulative variances captured by corresponding number of principal modes. Figure 1c shows the contribution of each NDM to data expanded via EOFs: the rates of presence of NDMs in different linear PCs are plotted. It is clear that each NDM captures its own fracture of PCs; essentially, only about twenty PCs participate in the nonlinear expansion: the subspace determined by them holds all nonlinearity we are able to extract from the data.

**Figure 2:**
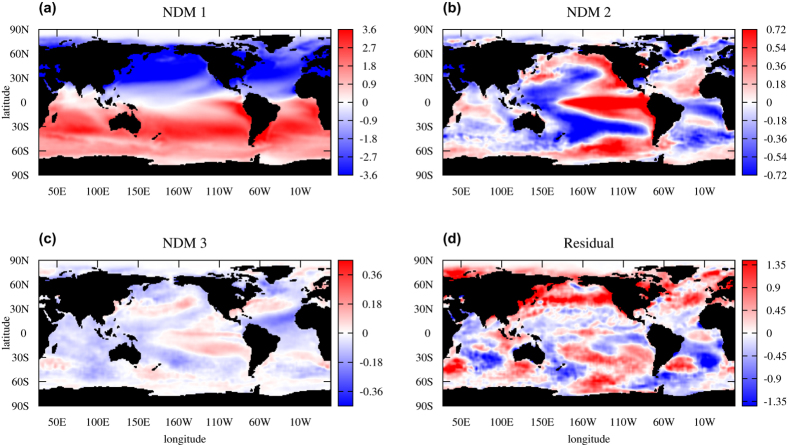
Representation of NDMs in data space. Snapshot corresponding to Jan. 1992 is shown. Captured fractions of SST are marked by color at each grid point. Modes 1, 2 and 3 are displayed on panels (**a**–**c**). Panel (**d**) shows the rest of the data that is not involved in the nonlinear expansion. Gnuplot (Copyright 1986–1993, 1998, 2004 Thomas Williams, Colin Kelley) was used to create the maps in this figure.

Since for every i^*th*^ NDM we have the corresponding function **F**_*i*_(.) mapping scalar values of *p*_*i*_(*t*) into the data space, it enables us to visualize the time series of NDMs on the geographic grid. Such a visualization is presented in the Supplementary Animation as well as snapshots (see Fig. 2a–c) for each of the three obtained NDMs separately. Also, in both this animation and Fig. 2d, the time series of the rest of data, which is not explained by three modes is shown. The first mode oscillates with approximately a 1-year period, clearly, this reflects the annual cycle in the data. The second and third modes have much larger time scales of order of ENSO and PDO variability; the second mode is apparently ENSO-related: it is excited with different signs during El-Niño and La-Niña events (see, for instance, Fig. 2 with the snapshot of the time series at time instant corresponding to El-Niño episode in 1992). The rest of the data (panels d in both Supplementary Animation and Fig. 2) behaves with shorter than 1 year character times; actually, it is treated as noise signal by our procedure.

### Analysis of principal modes

Let us look how the identified modes are manifested in different geographical regions and which climatic phenomena they reflect. We distinguished several SST-based indices corresponding to different regions in the Northern hemisphere, that have a significant impact on the global climate (see Table 1 for details): Nino 3.4 — ENSO SST-based index^1^, Pacific Decadal Oscillation (PDO) index derived as a projection of data on leading SST anomaly (SSTA) EOF in Northern Pacific area^29^, North Tropical Atlantic (NTA)^30^ and Indian Ocean Dipole (IOD)^31^ indices. Additionally, we introduced the North Atlantic (NA) index characterizing the temperature of the northern Atlantic ocean; it is defined as averaged SST anomalies over a certain rectangle area specified in Table 1.

**Figure 3:**
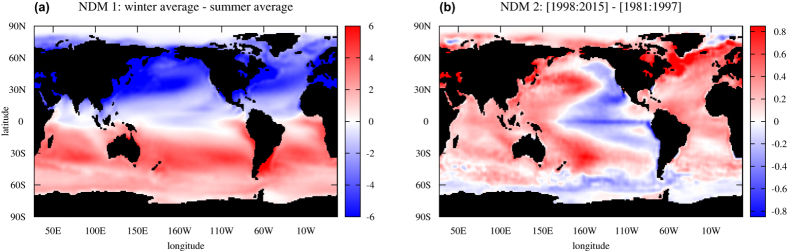
Spatial patterns explained by NDM 1 and NDM 2. (**a**) Annual cycle pattern: difference between SST captured by NDM 1 averaged over winter and summer months during the entire time interval of data. (**b**) Negative PDO pattern: difference between SST captured by NDM 2 averaged over the intervals [1998–2015] and [1981–1997]. Gnuplot (Copyright 1986–1993, 1998, 2004 Thomas Williams, Colin Kelley) was used to create the maps in this figure.

We calculated all these indices from each of three NDMs. The largest (first) NDM gives an almost periodical, slightly modulated signal in every NDM-based index (see Supplementary Figure S1). It corresponds to the annual cycle – the response of the climate to the 1-year periodic forcing. In doing so, the annual cycle is completely separated from data: there is no trace of harmonics of the 1-year periodical signal in the power spectra of the other modes and it is obtained in a natural way, without any periodical constraints in the procedure (see figures in Supplementary Figure S2). As a confirmation, Fig. 3a shows a clear global annual cycle pattern as a result of the difference of the average SST produced by the first NDM between summer and winter months during the whole period of observations.

**Figure 4:**
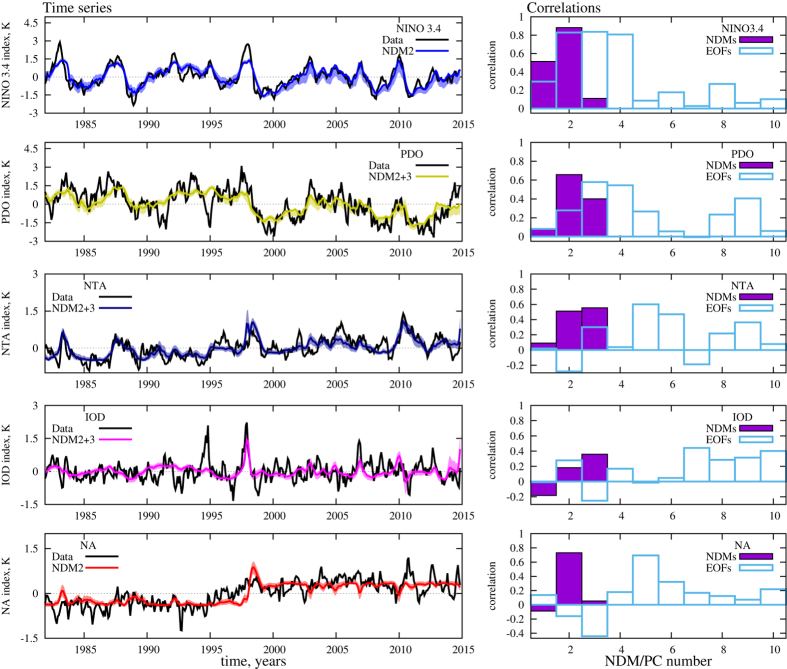
Reconstruction of climate indices enumerated in Table 1. Left panels: comparing original indices (black color) calculated from the SSTA field and those reconstructed from different combinations of NDM 2 and NDM 3. Bayesian confidence intervals of the reconstruction (see Supplementary Methods) are shown by shadow around each reconstructed index. Right panels: purple columns show correlations of certain index (see legend on figures) with different NDMs (number of NDM is on the abscissa). For comparison, correlations of the same indices with linear PCs – projections of data on EOFs – are shown by blue transparent columns. Gnuplot (Copyright 1986–1993, 1998, 2004 Thomas Williams, Colin Kelley) was used to create the graphs in this figure.

The left panels of Fig. 4 displays the NDM-based indices calculated from the combinations of NDMs yielding highest correlations with the corresponding original indices (black lines) derived from SSTA time series. Corresponding correlation coefficients are presented at the right column of Table 1. Purple columns on right panels of Fig. 4 show correlations of indices obtained from different NDMs (mean annual periodical signal was removed from the first NDM before calculations) with certain original index. For comparison, correlations of different linear PCs (after removing mean annual signal) with the original indices are shown as blue transparent columns on the same panels. We infer that the second NDM contributes, in a first place, to ENSO dynamics: it provides a high correlation of 0.88 with the original Nino 3.4 index. Note that the first NDM, which is responsible mainly for the annual cycle, also participates in ENSO variability having little more than 0.5 correlation with Nino 3.4 (see also Supplementary Figure S3). Moreover, it is seen from Fig. 1a that the hidden time series of the first and second NDMs are connected with each other: there are apparent jumps of both phase and amplitude in the almost periodic time series of the first NDM, which occurred during strong El-Niño events of 1982–83 and 1997–98. This fact can be interpreted as an evidence of a nonlinear interaction between ENSO and annual climate response – two modes with substantially different time scales. See also the comparison of ENSO indices derived from the first vs. second NDM in supplementary Figure S3. Anyhow, it is clear that the second NDM is dominant in ENSO variability; a similar situation takes place in the NA region: this NDM has a much higher contribution there than other NDMs. As for the two other considered indices – PDO and NTA – the third NDM becomes important as well: the second and the third NDMs together explain a substantial part of the SST variability in both regions. The Indian ocean dynamics is reflected less well: the three obtained NDMs give only a 0.38 correlation with the original IOD index.

However, contributions to the considered regions of linear PCs are spread over many PCs, as it can be seen from the right panels of Fig. 4. For instance, there are at least three PCs equally significant for ENSO dynamics as well as many PCs that contribute to PDO and Atlantic regions. Indeed, this is the result of linearity of the EOF-based rotation of data, leading to a mixing of different modes in the obtained components in case when nonlinear couplings are essential. Supplementary Figure S4 shows an advantage of our nonlinear approach over the traditional EOF decomposition to separate different time scales by reconstructed modes.

### Climate shift of 1997–1998

The most interesting event that plumped into our analyzed epoch is the 1997–1998 El-Niño episode – one of the strongest since records began (see the behavior of Nino 3.4 index on top panel of Fig. 4). There is an attractive hypothesis^6^ that this event plays a decisive role in both switching over the climate to a negative phase of PDO and a successive drastic change of the global warming scenario. Soon after the event a growth of the average atmospheric temperature had been suspended setting up the current hiatus in global warming^6^,^7^; before this the atmosphere was heated since the mid seventies during the positive PDO phase. It was shown^6^,^8^,^9^,^10^ that the significant part of heat goes into deep ocean during negative PDO that prevents the atmosphere from warming; at the same time melting ice and rising sea level is continued^32^ indicating that the global warming is in progress. But the regime of the warming was changed and, to all appearances, this is the result of internal dynamics of the climate system.

This climate shift manifests itself in our second NDM as a distinct jump in its hidden time series *p*(*t*) (Fig. 1a) on time interval comparable with one year. This is reflected in the time series of PDO and two Atlantic indices reconstructed from this NDM, as an abrupt change of averages (see corresponding panels of Fig. 4). Since this event is associated with a PDO phase change, we see how our second NDM, where the corresponding shift is observed, captures the PDO pattern. To this end, we calculated averages of SST captured by this single mode, over the period both before and after the shift, and then plotted the difference between these two patterns (Fig. 3b). The negative PDO pattern is manifested clearly in the Pacific region, that is in good correspondence with figure 9 from paper^6^, where similar differences are shown, but calculated from full surface temperature data. Thus, we can conclude that the second NDM, driven by only a single scalar time series, properly reflects the nature of the climate shift and confirms the hypothesis of the triggering role of the 1997–98 El-Niño episode^6^.

### Determining teleconnections

**Figure 5:**
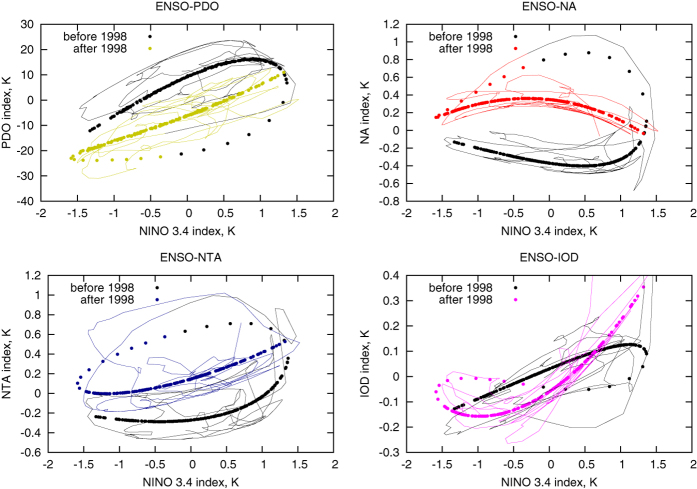
Couplings between ENSO and dynamics in other regions, revealed by NDMs. The dependencies derived from NDM 2 are shown by points, those reconstructed from superposition of NDM 2 and NDM 3 – by thin lines. Black color embraces the period before the 1997–1998 climate shift, other colors – after the shift. Colors after the shift are the same as in Fig. 4; they mark the coupling of ENSO and corresponding indices from that figure. Gnuplot (Copyright 1986–1993, 1998, 2004 Thomas Williams, Colin Kelley) was used to create the graphs in this figure.

Detecting principal curves in data space allows investigating teleconnections – the linkages of dynamics at widely-spaced regions of the globe (this is a typical notion for long-range connections in climatology^33^). Really, the second NDM captures about 7% of the whole observed SST variability. If we exclude from consideration the annual cycle, contained in the first NDM that holds about 78%, we see that the second NDM holds about 27% of SST anomalies (in this case the anomalies are regarded as data after subtraction of the first NDM). But this mode attaches the dynamics at all grid points to a one-dimensional curve and therefore, different points, and hence, different regions and different SST-based indices are coupled by this mode in a very simple way. In Fig. 5 (points) the curves derived from the second NDM become exhibited: they determine the dominant dependencies between the behavior in the Nino 3.4 region and other considered regions. They all consist of two branches – one corresponds to the period before the shift and the other – after the shift; note, that the shift led to significant changes in these curves. Adding the third NDM gives a bit spread dependencies, but the main shapes of them remain the same: thin lines in Fig. 5 denote couplings derived from the sum of the second and third NDMs which together hold more than 30% of SST anomalies.

## Discussion

In this work we expanded space-distributed global SST time series into a set of principal nonlinear modes. Each of these modes couples the dynamics at all grid points by a parametrically defined curve in data space. The proposed method uses *a priori* information about the dynamical nature of the reconstructed components by using certain restriction on the time scales inherent of the modes. The complexity of such nonlinear transformations as well as the characteristic time scale of every mode are both estimated to be relevant to available data: the Bayesian approach to optimal model selection is used. Moreover, the number of nonlinear modes we are able to resolve from data is also derived from a Bayesian criterion, that gives us a set of statistically significant nonlinear components driving the observed variability. The analysis of the 33-year time series of the SST field yielded three nonlinear modes underlying the dynamics, each of them is determined by single scalar time series. The most powerful one is associated with the annual cycle; it completely separates the 1-year oscillation from the rest of the data. The second mode describes a major part of ENSO-related variability. Eventually, combinations of the second and the third modes explain PDO, NTA and Northern Atlantic SST anomalies: they give more than 0.7 correlations with the corresponding indices.

The obtained expansion is interesting from the point of view of global climate analysis including a description of different phases of global warming regime. Really, the resulting NDMs reflect three main components of global climate variability, which have characteristic time scales less than the duration of the analyzed time series: annual cycle, ENSO mode and decadal mode associated with PDO behavior. In particular, the single second NDM correctly reproduces the climate shift of 1997–98–the change of PDO phase: the nature of the shift is clearly manifested in SST variability captured by this NDM. Further, if we consider the set of time series *p* driving the obtained NDMs as a source for phase variables of some dynamical subsystem governing the observed variability, it will be possible to construct the phase space in different ways. Figure 1d displays a projection consisting of three time series *p*_1_(*t*), *p*_2_(*t*), *p*_3_(*t*). The climate shift in this space looks like a transition between two areas. However, we analyzed here quite short time series, which includes only the single transition. Further expansion of longer data taking into consideration several such climate shifts in the past–including PDO transitions from a positive to a negative phase and back–would be the proper step on the way of constructing a dynamical model suitable for forecasting future events on decadal scales, which strongly affect global warming scenario. That is a challenging current problem in climate science: modern global climate models hardly predict internal decadal variability^6^, including current hiatus in global warming.

## Methods

According to Eq. (1), the main aim of the proposed expansion is to decompose multivariate (space-distributed) time series consisting of points **X**(*t*_*n*_) : = **X**^*n*^ into a set of principal modes, each of them is represented as a one-dimensional parametrically defined curve 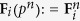. Below, we state briefly the main points of the method we suggest; more technical details can be found in the Supplementary Methods.

### Bayesian reconstruction

As in traditional EOF decomposition, we use a recursive procedure for a successive reconstruction of terms (modes) in Eq. (1). The first step is finding the leading mode Φ : = **F**_1_, having the largest contribution to data variance, by minimization of mean square deviations of residuals between data vectors **X**^*n*^ and mode values Φ^*n*^. The main problem here is the extremely high dimension of climate data defined on spatial grid, which makes such a minimization very hard computationally. Therefore, a preliminary data truncation would be reasonable. For this purpose we firstly rotate the data to the basis of EOFs which gives the set of PCs – the new variables **Y**^*n*^ = **V**^*T*^**X**^*n*^ – which are ranked in accordance with their variances (EOFs are the columns of the orthogonal matrix **V**). After such a rotation the main problem is to determine the subspace in the whole space of PCs, where the sought nonlinear curve **F**_*i*_(.) actually lies. Let us then construct the nonlinear transformation only in the space of the *d* first PCs, while the other PCs are treated as noise:


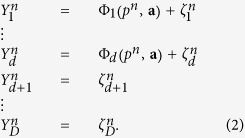


Here **a** are the unknown parameters in the Φ representation, *D* is the total number of PCs, *ζ* are the residuals, which are assumed to be Gaussian and uncorrelated. Thus, for reconstruction of this leading mode we should find proper values of both the latent variables *p*^*n*^ and the parameters **a**. Note that it is easy to show that in the special case of linear Φ this strictly corresponds to the traditional EOF rotation: minimization of squared residuals under condition |**a**| = 1 gives the largest EOF in the **a** vector and the corresponding PC in *p* time series.

In the framework of the Bayesian paradigm the cost-function used for learning model (2) is constructed in a form of a probability density function (PDF) of unknown variables under the condition of data **X**^1^, ..., **X**^*N*^: the learning model (2) means finding the global maximum of this PDF over unknown **a** and *p*^1^, ..., *p*^*N*^. This PDF can be expressed through the Bayes theorem:


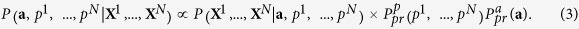


Here *P*(**X**^1^, ..., **X**^*N*^|**a**, *p*^1^, ..., *p*^*N*^) : = *L*(**a**, *p*^1^, ..., *p*^*N*^) is a likelihood function, i.e. the probability that the data **X** are obtained by both the model (2) with the parameters **a** and the series *p*^1^, ..., *p*^*N*^, and the subsequent rotation by matrix **V**. This function can be easily written under the assumption of normality and whiteness of noise *ζ* (see Supplementary Methods for details); its maximum is reached at minimal mean square errors of *ζ*. But the crucial point is the specification of a priori information, which reflects all desirable properties of the solution, in a form of prior probability densities of **a** and *p*^1^, ..., *p*^*N*^.

The main *a priori* assumption we suggest to use is the dynamical nature of the hidden mode *p* time series; in other words, we require every current point *p*^*n*^ to be connected with the previous one *p*_*n*−1_. Namely, we introduce a prior probability density for *p*^1^,..., *p*^*N*^ in the following form:


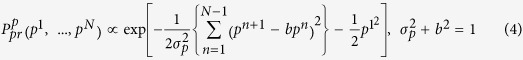


Actually, Eq. (4) determines a prior ensemble of *p* time series consisting of various realizations of colored noise so as most of them have an auto-correlation time 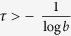. The second condition in (4) provides unit prior variance of each *p*^*n*^ ~ *N*(0, 1). Though the ensemble so defined is quite general, it provides an efficient restriction of the class of possible solutions by excluding from consideration short-scale signals.

Under the prior constraint (4) we introduced a representation of the functions Φ(.) in (2) as a superposition of polynomials Π_*i*_(*p*) which are mutually orthogonal in the probability measure defined by (4):


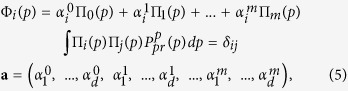


where *δ*_*ij*_ is Kronecker delta. Such a representation facilitates a model learning procedure substantially, since the problem becomes linear with respect to the parameters **a**. At the same time it allows an increasing model power by simply adding more orthogonal polynomials in (5). The idea how to set a prior PDF for the parameters of such a representation is quite apparent: it should be the most general, but permitting functions Φ_*i*_(.) to have *a priori* the same variances as the corresponding time series of PC *Y*_*i*_ (correlations between different PCs *Y*_*i*_ are zero, so we need not to care about covariances). Thus, it is reasonable to define this PDF as a product of Gaussian functions of each 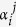 with variance 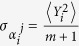, where 〈.〉 denotes average over time. The same restrictions of parameters were used, for instance, in^34^,^35^ regarding to external layer coefficients of artificial neural networks used for fitting an evolution operator.

### Optimal model selection

It is clear that the proposed method provides a solution that strongly depends on three parameters: *d* - the number of PCs involved in the nonlinear transformation, *m* - the number of orthogonal polynomials determining the degree of nonlinearity, and *τ* - the characteristic autocorrelation time of the reconstructed mode. All these values determine the complexity of the data transformation, and therefore should be relevant to the available statistics. For example, if we take a very large *m* and *τ* = 0, we would obtain the curve passing through every point 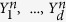, and our single mode *p*^1^, ..., *p*^*N*^ would capture the whole variability in the subspace of the *d* first PCs. But, indeed, in this case we would get an overfitted, statistically unjustified model. Thus, a criterion of optimality is needed allowing the selection of the best model from the model set defined by different values of (*d*, *m*, *τ*). We define optimality *E* through a Bayesian evidence function, i.e. the probability density of data **X**^1^, ..., **X**^*N*^ given the model (*d*, *m*, *τ*):


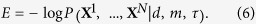


Actually, *E* is the minimal amount of information required for transferring the given series **Y**^1^, ..., **Y**^*N*^ by the model (*d*, *m*, *τ*); so, the same criterion could be derived in the framework of the minimal description length principle^36^). The model corresponding to the smallest E, or, which is the same, providing the maximum probability of reproducing the observed time series, is regarded to be optimal.

The Bayesian evidence function can be calculated by the integral of likelihood over all parameters **a** and latent variables *p*^1^, ..., *p*^*N*^ with the prior probability measure:


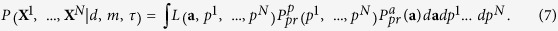


We calculate this integral by the Laplace method assuming that the main contribution comes from the neighborhood of the maximum of the integrand over all variables; see details in Supplementary Methods. The eventual optimality can be expressed in the following form:


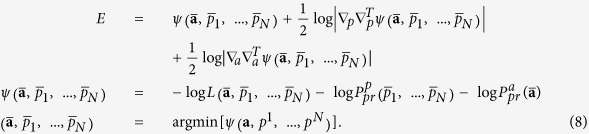


Here 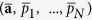 are estimated values corresponding to the minimum of the function *ψ*(.) – minus log of the cost-function; 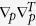 and 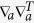 denote operators of second derivations over all *p*^*n*^ and **a** correspondingly. The last two terms in (8) penalize the model complexity; they provide the existence of an optimum of *E* over the model set. Thus, for estimating the optimality of the given model with (*d*, *m*, *τ*), we should (i) learn the model by finding the values 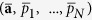 and then (ii) calculate penalty terms as determinants of *ψ*(.) second derivation matrices in the domains of parameters **a** and latent variables *p*^1^, ..., *p*^*N*^ in the point 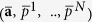.

### Main steps of the algorithm

The main steps of the proposed algorithm are the following.

1. Rotating given data 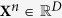 to PCs 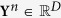: **Y**^*n*^ = **V**^*T*^**X**^*n*^; columns of the **V** matrix are EOFs of the **X** time series.
2. Finding the optimal model in the space of PCs **Y**. This step includes finding the optimal dimension *d* of the subspace for a nonlinear expansion, estimation of the optimal degree of nonlinearity *m* as well as characteristic autocorrelation time *τ* of the hidden mode. Here we also obtain concrete parameters 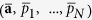 of the optimal mode.
3. Subtracting the obtained mode corresponding to the maximum of the cost-function from the data vectors: 
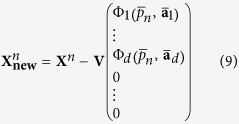
 The vector of the obtained mode image in the PC space is complemented by *D* − *d* zeros, since only *d* PCs are involved in the nonlinear transformation. In fact, at this step we set the new data vectors 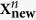 – the residuals between initial ones and values of the mode. After that we are going to find the next mode: the same procedure beginning from i.1 is applied to the new data 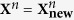.

The iteration of the procedure i.1–i.3 is stopped when we obtain time series *p* of the new mode equal to constant zero, or, equivalently, we find that the optimal degree *m* of polynomials (5) is equal to zero. It means that we cannot resolve any more nonlinearity in the data, and the noise is most probably significant; in other words, all the best we can further do is traditional EOF decomposition of the residuals. In particular, the expansion of SST time series into three NDMs presented in the current paper gives *d* = 5, *m* = 3 for the first NDM, *d* = 4, *m* = 6 for the second and *d* = 8, *m* = 8 for the third. Eventually, the entire nonlinear expansion of data can be written as follows:


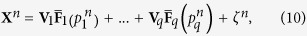


where *q* is the total number of obtained NDMs. Note that each of the NDMs is defined in its own subspace, which is rotated relative to the initial data space: the orthogonal matrices **V**_*i*_ are different for different NDMs.

## Additional Information

**How to cite this article**: Mukhin, D. *et al.* Principal nonlinear dynamical modes of climate variability. *Sci. Rep.* **5**, 15510; doi: 10.1038/srep15510 (2015).

## Supplementary Material

Supplementary Information

Supplementary Animation

## Figures and Tables

**Table 1:** Indices derived from SST field reconstructed from obtained NDMs.

Index	Region	Description	Correlation with SSTA-based index
Nino 3.4	[5°S–5°N; 190°E–120°W]	Averaged data	0, 88
PDO	[20°N–60°N; 140°E–120°W]	Projection on largest SSTA EOF	0, 75
NTA	1. [6°N–18°N; 60°W–20°W] 2. [6°N–10°N; 20°W–10°W]	Averaged data over both 1 and 2 regions	0, 74
NA	[45°N–70°N; 60°W–0°W]	Averaged data	0, 73
IOD	1. [10°S–10°N; 50°E–70°E] 2. [0°N–10°N; 90°E–110°E]	Difference between averaged data in regions 1 and 2	0, 38

Correlations of the indices with “true” ones, calculated from full SSTA field, are presented in the last column.
