# Supplementary material for: Principal nonlinear dynamical modes of climate variability
Source: Sci Rep. 2015 Oct 22;5:15510. doi: 10.1038/srep15510 (PMC5155699; doi:10.1038/srep15510)
Supplement: Supplementary Information [file srep15510-s1.pdf]

Supplementary Material

Principal nonlinear dynamical modes of climate variability

Dmitry Mukhin[1,*], Andrey Gavrilov[1], Alexander Feigin[1,2], Evgeny Loskutov[1], and Juergen Kurths[3,1]

[1]Institute of Applied Physics of the Russian Academy of Sciences, 603950 Nizhny Novgorod, Russia
[2]Lobachevsky State University of Nizhni Novgorod, 603950 Nizhny Novgorod, Russia
[3]Potsdam Institute for Climate Impact Research, 14412 Potsdam, Germany
[*]mukhin@ipfran.ru

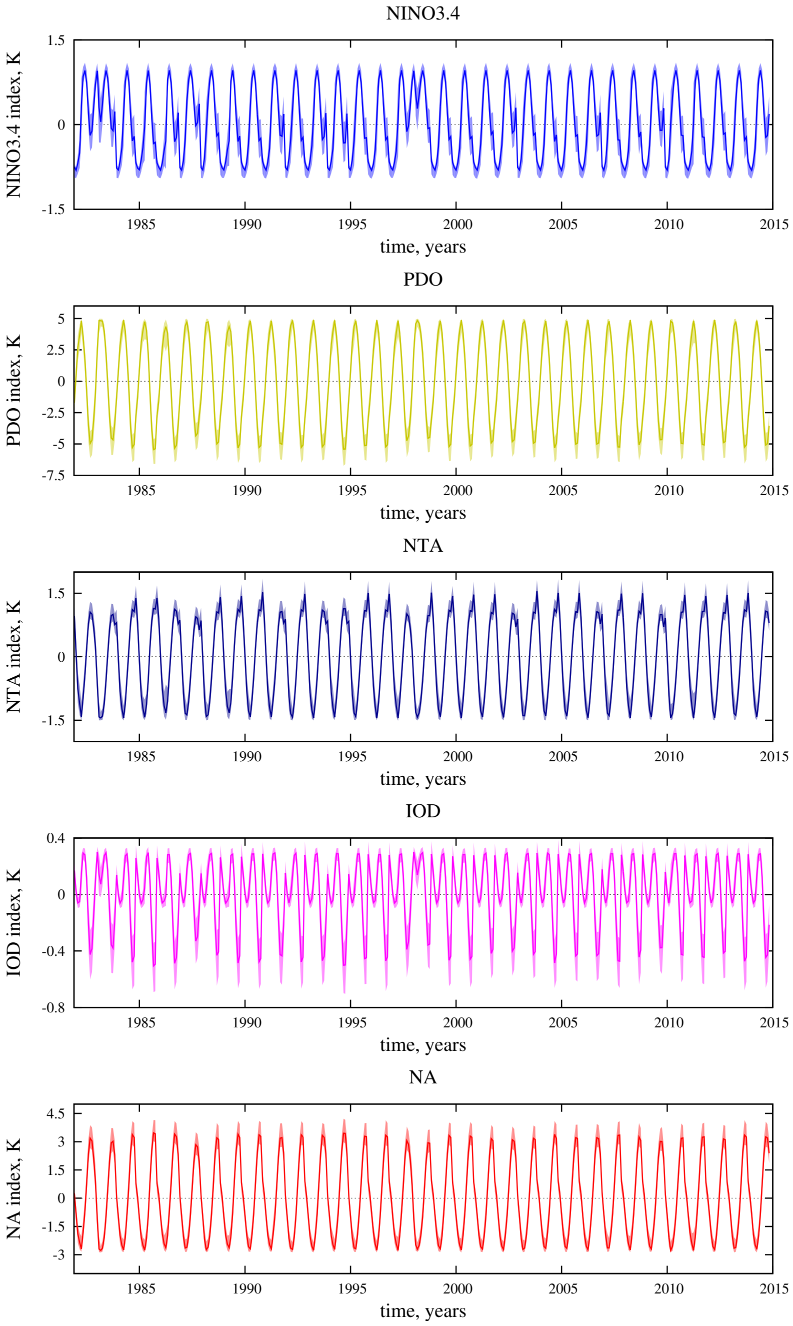

Figure S1. Time series of indices calculated in different regions as defined in the table 1 (the main text) from the global SST field reconstructed from the NDM 1. Bayesian 95% confidence intervals are provided in shadowed colors. Annual period dominates in every index. Gnuplot (Copyright 1986 - 1993, 1998, 2004 Thomas Williams, Colin Kelley) was used to create the graphs in this figure.

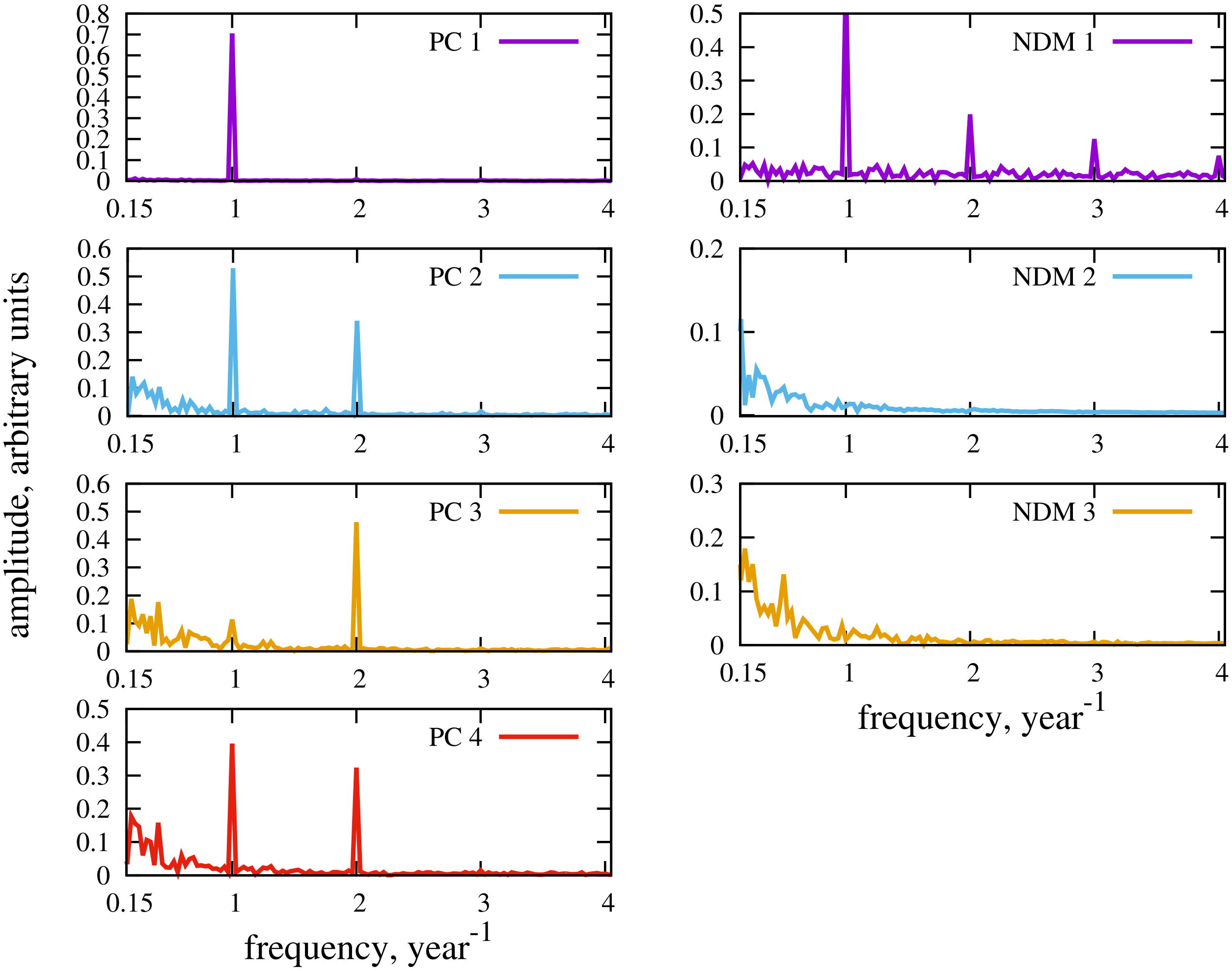

Figure S2. PCs (left panels) and NDM hidden time series (right panels) Fourier spectra amplitudes (from global SST data). Annual cycle is entirely captured by the NDM 1, while spread among many EOFs. Gnuplot (Copyright 1986 - 1993, 1998, 2004 Thomas Williams, Colin Kelley) was used to create the graphs in this figure.

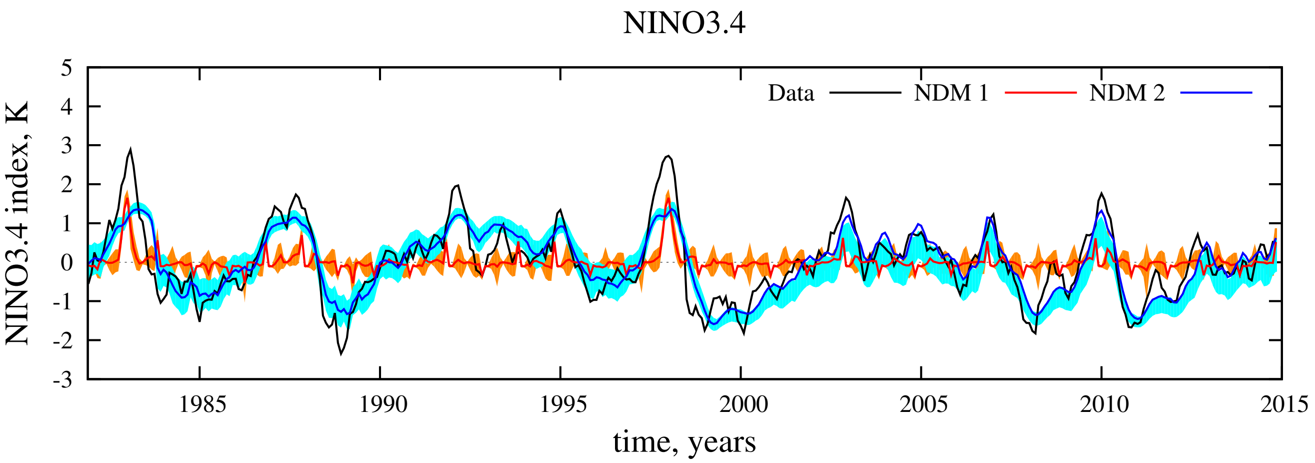

Figure S3. Comparing Nino3.4 time series: red – Nino 3.4 index derived from the SST fields reconstructed from NDM 1 (with subtracted mean annual signal) with its 95% Bayesian confidence interval; blue – Nino 3.4 index derived from the SST field reconstructed from NDM 2 with its 95% Bayesian confidence interval; black – the original SSTA-based Nino3.4 data. The two strongest El-Nino events are partially captured by the NDM 1, the others are not. Gnuplot (Copyright 1986 - 1993, 1998, 2004 Thomas Williams, Colin Kelley) was used to create the graphs in this figure.

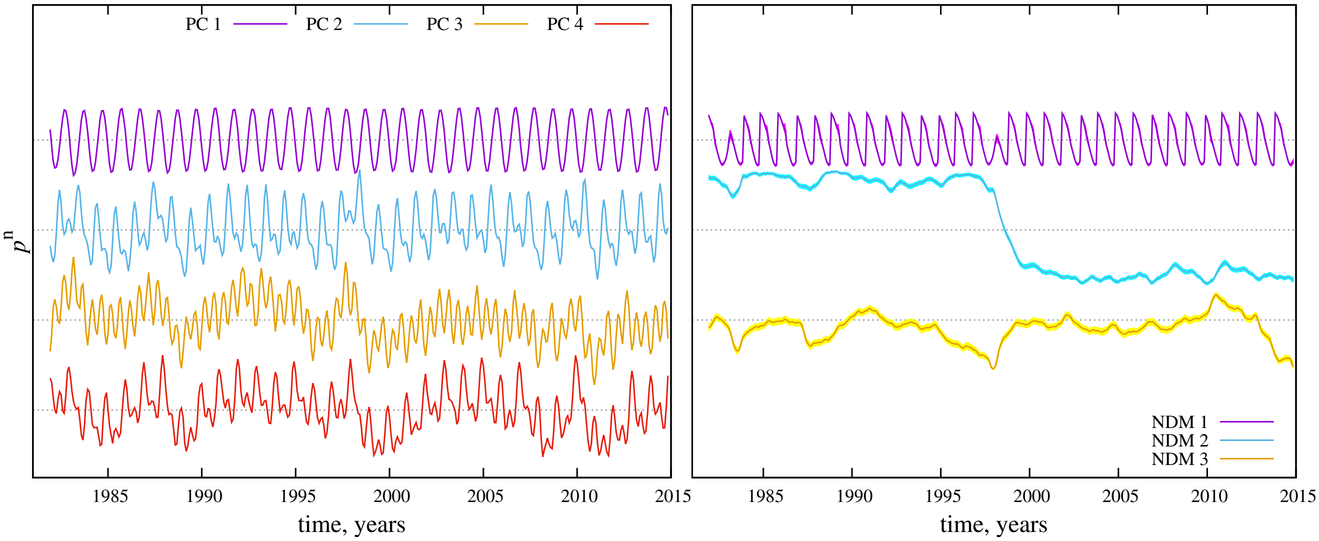

Figure S4. Comparing the PCs produced by EOF decomposition (normalized to equal standard deviation) and hidden time series of NDMs (line thickness reflects the 95% Bayesian confidence interval), both for global SST data. The time scales are much better separated by the NDMs. Gnuplot (Copyright 1986 - 1993, 1998, 2004 Thomas Williams, Colin Kelley) was used to create the graphs in this figure.

Caption of Supplementary Animation

Representation of NDMs in data space (see text of the paper). Captured fractions of SST are marked by color at each grid point. Modes 1,2 and 3 are displayed on the top and left bottom panels. The right bottom panel shows the rest of the data that is not involved in the nonlinear expansion. Gnuplot (Copyright 1986 - 1993, 1998, 2004 Thomas Williams, Colin Kelley) was used to create this animation.

Supplementary methods

1. Nonlinear dynamical modes decomposition

Let the D-dimensional time series $\mathbf{X}^n$ be the result of an observation:

$$\{\mathbf{X}^n = \mathbf{X}(t^n)\}_{n=1}^{N}, \quad \mathbf{X}^n \in \mathbb{R}^D \tag{S1}$$

with equal distance between the time moments t^n enumerated by the index n and data length N. Note that hereinafter spatially distributed time series are understood as a particular case of multidimensional time series, when the coordinates of the vector $\mathbf{X}^n$ are given by enumerating spatial nodes.

In order to catch nonlinear interactions described in the main text we suggest an iterative procedure of data decomposition via the sum:

$$\begin{aligned} &\mathbf{X}^n = \mathbf{F}_1(\mathbf{a}_1, p_1^n) + \mathbf{F}_2(\mathbf{a}_2, p_2^n) + \ldots; \\ &n = 1, \ldots, N \end{aligned} \tag{S2}$$

Here $\mathbf{F}_i(\mathbf{a}_i, \cdot) : \mathbb{R} \to \mathbb{R}^D$ is a time-independent nonlinear mapping parameterized by $\mathbf{a}_i \in \mathbb{R}^M$, and p_i^n is its argument which depends on the discrete time n. Each term in the form of $\mathbf{F}(\mathbf{a}, p^n)$ can be physically interpreted as a nonlinear multidimensional (or, particularly, spatially distributed) response of the system to the hidden scalar signal p^n. The terms $\mathbf{F}_i(\mathbf{a}_i, p_i^n)$ governed by the scalar signals p_i will be called nonlinear dynamical modes (NDMs), which are found with specific prior assumptions by the method described below.

A geometrical interpretation of a NDM is also useful. Generally, the parameter $\mathbf{a}$ of the function $\mathbf{F}$ defines a nonlinear curve in $\mathbb{R}^D$. Then the time series $p^1, ..., p^N$ are just coordinates of N points along such a curve. These points are specific projections of the observed data (see Fig.S5 for illustration).

The iterative scheme of the decomposition is constructed as follows. On the k-th iteration ($k = 1, 2, \ldots$) we fit the term $\mathbf{F}(\mathbf{a}, p^n)$ optimally into the residual $\mathbf{X}^n - \sum_{i=1}^{k-1} \mathbf{F}_i(\mathbf{a}_i, p_i^n)$. Here $\mathbf{a}$ and $p^1, \ldots, p^N$ are the fitted parameters. The particular parameterization of the model to fit as well as a cost function and a criterion of optimality of the fitting are described in detail below. As a result of the k-th iteration, we obtain the k-th NDM.

It is interesting that the classic empirical orthogonal functions (EOF) decomposition[1] can be represented via the sum (S2) with $\mathbf{F}_i(\mathbf{a}_i, p_i^n) = \mathbf{a}_i \cdot p_i^n$ and fitting restricted by the condition of a fixed length $\|\mathbf{a}\| = 1$. Thus, the EOF decomposition can be regarded as a linear case of the decomposition (S2).

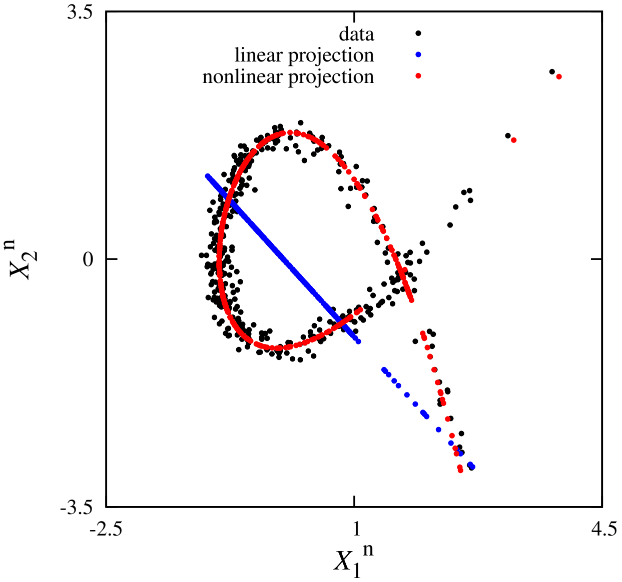

Figure S5. Schematic example of the idea of nonlinear mode (nonlinear projection of the data on the curve) compared to EOF (orthogonal projection of the data on the line).

In a general case the key problem is finding an optimal (probably nonlinear) structure of parameterization for each mode $\mathbf{F}(\mathbf{a},\cdot)$; it depends on the dataset structure and the duration of the observed time series. This problem can be solved within a Bayesian framework.

1.1 NDM parameterization

A Bayesian formulation of the optimal fitting problem assumes that we have a set of models which can be fit into the given data (this procedure will be called "model learning"), and we should choose the most useful (optimal) model from this set. In this section we are going to write explicitly the functional form which we use to define the set of models on each iteration of the above-described scheme.

Let us denote the residuals given on a particular iteration, again, as the time series of D-dimensional column-vectors $\mathbf{X}^1,\ldots,\mathbf{X}^N$ (following the notation at step 3 of the algorithm given in the main text).

First of all, we define the rotation of the dataset $\mathbf{X}^1,\ldots,\mathbf{X}^N$ to its principal components (PCs), i.e. the preliminary EOF decomposition:

$$\mathbf{Y}^n = \mathbf{V}^T\mathbf{X}^n \tag{S3}$$

Here $\mathbf{V}$ is an orthonormal matrix whose columns are eigenvectors of the given data covariance matrix:

$$\mathbf{C}_X = \frac{1}{N}\sum_{i=1}^{N}\mathbf{X}^n\mathbf{X}^{nT} \tag{S4}$$

The components Y_i^n of the column-vector $\mathbf{Y}^n$ are ordered such that their variances, i. e. eigenvalues of $\mathbf{C}_X$, decrease with $i=1,\ldots,D$. The rotation (S3) is especially appropriate for the case when the initial data is spatially distributed and has therefore many nodes with similar dynamics. It filters the directions in the D-dimensional space by their significance and allows us to include the EOF-based truncation into the model in a natural way.

Now let us define the truncated column-vector $\mathbf{Y}_d^n$:

$$\mathbf{Y}_{\mathbf{d}}^n = \begin{pmatrix} Y_1^n \\ \vdots \\ Y_d^n \end{pmatrix} \tag{S5}$$

and introduce the following form of the model:

$$\begin{cases} \mathbf{Y}_{\mathbf{d}}^n = \mathbf{\Phi}(\mathbf{a},p^n) + \sigma_\Phi\cdot\boldsymbol{\xi}_{\mathbf{d}}^n \\ Y_{d+1}^n = \sigma_{d+1}\cdot\xi_{d+1}^n \\ \cdots \\ Y_D^n = \sigma_D\cdot\xi_D^n \end{cases} \tag{S6}$$

Here we make the assumption that the d leading EOFs are nonlinearly coupled by the function $\mathbf{\Phi}(\mathbf{a},p^n)$ governed by the scalar time series $\mathbf{p}=(p^1,\ldots,p^N)$, while the residuals and the other EOFs are modelled by Gaussian delta-correlated processes $\boldsymbol{\xi}_{\mathbf{d}}^n \sim N(0,\mathbf{I}_{\mathbf{d}})$ (here $\mathbf{I}_{\mathbf{d}}$ is an identity matrix $d\times d$) and $\xi_{d+1}^n \sim N(0,1),\ldots,\xi_D^n\sim N(0,1)$ with intensities collected in the vector:

$$\boldsymbol{\sigma} = \begin{pmatrix} \sigma_\Phi \\ \sigma_{d+1} \\ \vdots \\ \sigma_D \end{pmatrix} \tag{S7}$$

In order to define a nonlinear function of p^n in (S6), we use a polynomial parameterization as it is one of the universal approximators for nonlinear dependencies:

$$\mathbf{\Phi}(\mathbf{a},p) = \sum_{i=0}^{m}\boldsymbol{\alpha}^i\cdot\Pi_i(p) \tag{S8}$$

Here the vector $\mathbf{a}$ is formed by the components of all $\boldsymbol{\alpha}^i\in\mathbb{R}^d$:

$$\mathbf{a} = \left(\alpha_1^0,...,\alpha_d^0;\alpha_1^1,...,\alpha_d^1;...;\alpha_1^m,...,\alpha_d^m\right) \tag{S9}$$

An infinite basis of polynomials $\{\Pi_0, \Pi_1, ..., \Pi_m, ...\}$ is fixed in (S8), with $\Pi_i(p)$ being a Hermitian polynomial of degree i normalized to unity (convenience of such choice of basis will be seen in further sections).

A deterministic part of such model at particular values of parameters will be considered as NDM (as it follows from (S3) and (S6)):

$$\mathbf{F}(\mathbf{a}, p^n) = \mathbf{V_d} \cdot \mathbf{\Phi}(\mathbf{a}, p^n) \tag{S10}$$

Here $\mathbf{V_d}$ is the matrix with d columns which are d leading EOFs corresponding to the truncated PCs (S5).

1.2 Bayesian NDM learning

The Bayesian approach to model learning is based on the analysis of the posterior parameter probability distribution $P(\mathbf{a}, \mathbf{p}, \boldsymbol{\sigma} | \mathbf{X}^1, \ldots, \mathbf{X}^N)$ of the model under the condition that the dataset $\mathbf{X}^1, \ldots, \mathbf{X}^N$ was observed. The estimation of this distribution is given by the Bayes theorem:[2]

$$P(\mathbf{a}, \mathbf{p}, \boldsymbol{\sigma} | \mathbf{X}^1, \ldots, \mathbf{X}^N) = \frac{1}{A(\mathbf{X}^1, \ldots, \mathbf{X}^N)} \cdot P(\mathbf{X}^1, \ldots, \mathbf{X}^N | \mathbf{a}, \mathbf{p}, \boldsymbol{\sigma}) \cdot P_{pr}(\mathbf{a}, \mathbf{p}, \boldsymbol{\sigma}) \tag{S11}$$

Here the normalization term $A(\mathbf{X}^1, \ldots, \mathbf{X}^N)$ is independent of the model parameters. The second term is the likelihood fuction which can be written for any parameterization of $\mathbf{f}$ directly from (S3), (S6):

$$P(\mathbf{X}^1, \ldots, \mathbf{X}^N | \mathbf{a}, \mathbf{p}, \boldsymbol{\sigma}) = \prod_{n=1}^{N} P(\mathbf{X}^n | \mathbf{a}, p^n, \boldsymbol{\sigma}) = \prod_{n=1}^{N} P(\mathbf{Y}^n | \mathbf{a}, p^n, \boldsymbol{\sigma}) \tag{S12}$$

with

$$P(\mathbf{Y}^n | \mathbf{a}, p^n, \boldsymbol{\sigma}) = \left(2\pi\sigma_\Phi^2\right)^{-\frac{d}{2}} \cdot \exp\left(-\frac{\left\|\mathbf{Y}_\mathbf{d}^n - \mathbf{\Phi}(\mathbf{a}, p^n)\right\|^2}{2\sigma_\Phi^2}\right) \times \prod_{k=d+1}^{D} \left[\left(2\pi\sigma_k^2\right)^{-\frac{1}{2}} \cdot \exp\left(-\frac{|Y_k^n|^2}{2\sigma_k^2}\right)\right] \tag{S13}$$

Here the operation $\|\cdot\|$ denotes the euclidean norm in $\mathbb{R}^d$.

The last term in (S11) is the prior probability distribution $P_{pr}(\mathbf{a}, \mathbf{p}, \boldsymbol{\sigma})$ which does not depend on the observed data, since it is a property of the model.

The parameters $(\bar{\mathbf{a}}, \bar{\mathbf{p}}, \bar{\boldsymbol{\sigma}})$ which maximize (S11) are considered here as the result of model learning. Technically, maximization of (S11) is equivalent to minimizing the following cost function φ by $(\mathbf{a}, \mathbf{p}, \boldsymbol{\sigma})$:

$$\varphi(\mathbf{a}, \mathbf{p}, \boldsymbol{\sigma}) = -\log\left[P(\mathbf{X}^1, \ldots, \mathbf{X}^N | \mathbf{a}, \mathbf{p}, \boldsymbol{\sigma}) P_{pr} \cdot (\mathbf{a}, \mathbf{p}, \boldsymbol{\sigma})\right] \to \min \tag{S14}$$

To solve the problem (S14),we set the prior distribution as factorized by $\mathbf{a}$, $\mathbf{p}$ and $\boldsymbol{\sigma}$:

$$P_{pr}(\mathbf{a}, \mathbf{p}, \boldsymbol{\sigma}) = P_{pr}^{a}(\mathbf{a}) \cdot P_{pr}^{p}(\mathbf{p}) \cdot P_{pr}^{\sigma}(\boldsymbol{\sigma}) \tag{S15}$$

In Bayesian modeling the choice of the relevant prior distributions is very important: it should be adequate to the problem from both mathematical and physical points of view, as well as the choice of parameterization.

Prior distribution of $\boldsymbol{\sigma}$ The problem (S14) without any prior requirements is extremely well-posed by σ as a one-dimensional minimization problem. Physically it means that for any form of the mode one can easily calculate the deviation of the data from the mode.

That is why it is sufficient to let P_{pr}^{σ} be a non-zero constant on a finite area covering all expected values of $\boldsymbol{\sigma}$:

$$P_{pr}^{\sigma}(\boldsymbol{\sigma}) = const \tag{S16}$$

The function (S14) under the condition (S16) has a unique minimum by $\boldsymbol{\sigma}$ which can be explicitly found at any $(\mathbf{a}, \mathbf{p})$:

$$\boldsymbol{\sigma}_{min}(\mathbf{a}, \mathbf{p}) = \begin{pmatrix} \frac{1}{Nd}\sum_{n=1}^{N}\left\|\mathbf{Y}_\mathbf{d}^n - \mathbf{\Phi}(\mathbf{a}, p^n)\right\|^2 \\ \frac{1}{N}\sum_{n=1}^{N}\left|Y_{d+1}^n\right|^2 \\ \vdots \\ \frac{1}{N}\sum_{n=1}^{N}|Y_D^n|^2 \end{pmatrix} \tag{S17}$$

Since the definition (S10) of a NDM does not depend on $\boldsymbol{\sigma}$, it is convenient to exclude this parameter from the model (S6) defining $\boldsymbol{\sigma}$ as (S17). Such a model depends only on the parameters $(\mathbf{a},\mathbf{p})$ with the same prior distributions $P^a_{pr}(\mathbf{a})$ and $P^p_{pr}(\mathbf{p})$, its likelihood is derived from (S12)-(S13) and denoted in the main text as $L(\mathbf{a},p^1,\ldots,p^N)$:

$$L(\mathbf{a},\mathbf{p}) = P\left(\mathbf{X}^1,\ldots,\mathbf{X}^N|\mathbf{a},\mathbf{p},\boldsymbol{\sigma}_{min}(\mathbf{a},\mathbf{p})\right) \tag{S18}$$

This model has the following cost function to be minimized over $(\mathbf{a},\mathbf{p})$:

$$\psi(\mathbf{a},\mathbf{p}) = -\log\left[L(\mathbf{a},\mathbf{p})\cdot P^a_{pr}(\mathbf{a})\cdot P^p_{pr}(\mathbf{p})\right] \to \min \tag{S19}$$

Obviously, the problems (S14) and (S19) have the same solution $(\bar{\mathbf{a}},\bar{\mathbf{p}})$, i. e. the results of learning are equal for both models (with and without $\boldsymbol{\sigma}$). It can be shown also, that the results of Bayesian optimization described in sec. 1.3 are not affected by $\boldsymbol{\sigma}$ exclusion in the case $N >> 1$.

Prior distribution of the hidden time series p We expect that the hidden signal $\mathbf{p}$ is associated with the hidden dynamical system, i. e. the time sequence and the evolution law in $\mathbf{p}$ are important. At the same time we do not know the evolution law of the hidden system before the decomposition. In order to include our preferences into the model, we use the simplest evolution law differing from white noise as a prior information:

$$p^{n+1} = b\cdot p^n + \sigma_p\cdot\eta^n \tag{S20}$$

Here $\eta^n \in \mathbb{R}$ is a Gaussian delta-correlated random process. Equation (S20) is a linear stochastic operator (autoregressive process of first order) with two parameters b and σ_p. In general, it can be thought of making smooth signals more preferable. Indeed, the time scale of the autocorrelation function for the process (S20) is determined by b:

$$\tau = -\frac{1}{\ln b} \tag{S21}$$

Also, to remove an obvious degeneracy connected with linear stretching of the coordinate p along the curve and corresponding changes in the parameters $\mathbf{a}$ of the curve, we fix the amplitude of the process (S20) to be unity:

$$b^2 + \sigma_p^2 = 1 \tag{S22}$$

To specify $P^p_{pr}(\mathbf{p})$ we need to specify the distribution of p^1 initializing the process (S20). Naturally, it is supposed to be equal to the invariant measure P_{inv} of the process (S20) under the condition (S22):

$$P^p_{pr}\left(p^1\right) = P_{inv}\left(p^1\right) \tag{S23}$$

with

$$P_{inv}(p) = (2\pi)^{-\frac{1}{2}}\exp\left[-\frac{1}{2}|p|^2\right] \tag{S24}$$

Finally, from (S20)-(S24) we get the following prior distribution of $\mathbf{p}$:

$$\begin{aligned} &P^p_{pr}(\mathbf{p}) = (2\pi)^{-\frac{1}{2}}\exp\left[-\tfrac{1}{2}\left|p^1\right|^2\right]\times \\ &\quad\times\left(2\pi\sigma_p^2\right)^{-\frac{N-1}{2}}\exp\left[-\tfrac{1}{2\sigma_p^2}\textstyle\sum_{n=1}^{N-1}\left|p^{n+1}-b\cdot p^n\right|^2\right] \\ &b = \exp(-1/\tau) \\ &\sigma_p = \sqrt{1-\exp(-2/\tau)} \end{aligned} \tag{S25}$$

Thus, the sequence $\mathbf{p}$ corresponds to the process (S20) in the steady state. Actually, the distribution (S25) is normal with the inverse covariance matrix $\mathbf{B}$:

$$P^p_{pr}(\mathbf{p}) = \sqrt{\det\left(\frac{\mathbf{B}}{2\pi}\right)}\exp\left[-\frac{1}{2}\mathbf{p}^T\mathbf{B}\mathbf{p}\right] \tag{S26}$$

Here $\mathbf{p}$ is assumed as a column-vector, $\mathbf{B}$ is symmetric and tridiagonal and depends only on the free parameter τ.

Mathematically (S26) tries to solve the general problem of degeneracy among an infinite number of possible coordinates introduced on the same curve. It is especially important when the curve has a high curvature or self-intersections. Different coordinates can provide different, more or less “good”, hidden time series $\mathbf{p}$ which we evaluate using (S26).

Prior distribution of the parameters a Despite the condition (S22), the distribution (S26) does not exclude infinitely small variances of the time series **p** and corresponding degeneracy. Therefore the aim of the prior distribution of **a** is to do this job, as well as restrict the set of curves described by **f**.

We take the prior distribution of **a** in a Gaussian form with the covariance matrix **K**:

$$P_{pr}^{a}(\mathbf{a})=\frac{1}{\sqrt{\det(2\pi\mathbf{K})}}\exp\left[-\frac{1}{2}\mathbf{a}^{T}\mathbf{K}^{-1}\mathbf{a}\right] \quad \text{(S27)}$$

Here **a** is assumed as a column-vector.

The Gaussianity of (S27), together with the linearity of (S8) by **a**, provides an easy numerical implementation and calculation time advantages. At the same time, Gaussian distribution is a very general and popular way to specify a preferred area in the parameter space.

Note that the basis of normalized Hermitian polynomials in (S8) has the orthonormality property in terms of scalar product weighted with the invariant measure (S24) (by definition of Hermitian polynomials):

$$\left\langle \Pi_i\cdot\Pi_j\right\rangle_{P_{inv}}=\delta_{ij} \quad \text{(S28)}$$

with

$$\delta_{ij}=\begin{cases}1, & if \quad i=j\\ 0, & if \quad i\neq j\end{cases} \quad \text{(S29)}$$

This property is the reason of the Hermitian polynomials basis choice: different terms in (S8) are based on orthogonal polynomials.

In order to provide the regularizing properties of (S27) described above and taking into account the property (S28), we use the prior matrix **K** which:

(i) makes the covariance matrix of (S8) *a priory* equal to the covariance matrix of the input data $\mathbf{\Lambda_d}=\left\langle \mathbf{Y}_{\mathbf{d}}^{n}\mathbf{Y}_{\mathbf{d}}^{nT}\right\rangle_n$;

(ii) holds (S27) invariant under rotations of the orthonormal basis $\Pi_0,\ldots,\Pi_m$.

These conditions uniquely define the matrix **K** as the following matrix with the size $d\cdot(m+1)$:

$$\mathbf{K}=\frac{1}{m+1}\begin{pmatrix}\mathbf{\Lambda_d} & \ldots & 0\\ \vdots & \ddots & \vdots\\ 0 & \ldots & \mathbf{\Lambda_d}\end{pmatrix} \quad \text{(S30)}$$

Note that $\mathbf{\Lambda_d}$ is a diagonal matrix with variances $\lambda_k=\frac{1}{N}\sum_{n=1}^{N}\left|Y_k^n\right|^2$ of the first d PCs on the diagonal (it follows from our definition of $\mathbf{Y}_{\mathbf{d}}^{n}$).

Thus, taking into account (S30) we can write the prior distribution (S27) in the following form:

$$P_{pr}^{a}(\mathbf{a})=\prod_{i=0}^{m}\prod_{k=1}^{d}\frac{1}{\sqrt{2\pi\lambda_k/(m+1)}}\exp\left(-\frac{\left|\alpha_k^i\right|^2}{2\lambda_k/(m+1)}\right). \quad \text{(S31)}$$

1.3 Bayesian optimal NDM selection

In the scope of sec. 1.1 and sec.1.2 we have a set of models defined by triplets of free control parameters, or hyperparameters: the number d of PCs , nonlinearly coupled by the model in (S6); the degree of the nonlinearity m of the polynomial in (S8); and the prior time scale τ of the hidden time series **p** in (S25). Obviously, every particular triplet (d,m,τ) provides a set of various data generators (S6) with a prior measure on this set, and there is a problem of choosing the "best" triplet.

Within the Bayesian framework we evaluate the usefulness of the triplet (d,m,τ) for describing a given dataset as the probability of the corresponding model to generate this dataset:

$$P(\mathbf{X}^1,\ldots,\mathbf{X}^N|d,m,\tau)=\int_{\mathbf{a},\mathbf{p}}L(\mathbf{a},\mathbf{p})P_{pr}^{a}(\mathbf{a})P_{pr}^{p}(\mathbf{p}) \quad \text{(S32)}$$

Here the prior distributions P_{pr}^{a}, P_{pr}^{p} and the likelihood L under the integral are assumed for the hyperparameters (d,m,τ). The quantity (S32) is called Bayesian evidence, or marginal likelihood of the model.[3]

The optimal NDM is defined as (S10) with the parameters $(\bar{\mathbf{a}},\bar{\mathbf{p}})$ solving the problem (S19) for the triplet of hyperparameters $(\bar{d},\bar{m},\bar{\tau})$ maximizing the evidence (S32). This optimality criterion is completely equivalent to the minimum description length

principle (MDL)[4,5] for model selection in information theory, which defines an optimal model as one coding the observed data with the minimal amount of information E:

$$E(d,m,\tau) = -\log P(\mathbf{X}^1,\ldots,\mathbf{X}^N|d,m,\tau) \to \min. \tag{S33}$$

In general, the estimation of (S33) is a very hard problem. There are several methods of numerical approximation of an integral like (S32): the Laplace approximation,[6] the Gibbs sampling,[7] the EM algorithm.[8] We use the Laplace approximation (in the same way as in[9] for latent variables). It is especially approved here because of the Gaussianity of the likelihood (S12) by $\mathbf{a}$ in case of the polynomial parameterization (S8) and Gaussianity of the prior distribution by $\mathbf{p}$ (S26) and by $\mathbf{a}$ (S27). The expression for the cost function (S33) obtained by this method is:

$$E(d,m,\tau) \cong \psi(\bar{\mathbf{a}},\bar{\mathbf{p}}) + \frac{1}{2}\log\det\left(\frac{1}{2\pi}\boldsymbol{\nabla}\boldsymbol{\nabla}^T\psi\right). \tag{S34}$$

Here the function ψ is taken for the hyperparameters triplet (d,m,τ), and $\boldsymbol{\nabla}\boldsymbol{\nabla}^T\psi$ is the matrix of the second derivatives of ψ by $(\mathbf{a},\mathbf{p})$ at the minimum $(\bar{\mathbf{a}},\bar{\mathbf{p}})$.

Actually, the first term in (S34) is the minimum of the cost function (S19). The second term, generally, is a penalty for the complexity of the model. The computation of $\det\left(\frac{1}{2\pi}\nabla\nabla^T\psi\right)$ in our case can be additionally simplified at $N >> 1$ due to the facts below.

First, the elements of the matrix $\nabla_{\mathbf{a}}\nabla_{\mathbf{p}}{}^T\psi$ are N times smaller than the elements of $\nabla_{\mathbf{a}}\nabla_{\mathbf{a}}{}^T\psi$ and d times smaller than the elements of $\nabla_{\mathbf{p}}\nabla_{\mathbf{p}}{}^T\psi$. It follows that we can neglect the contribution of the mixed derivatives $\nabla_{\mathbf{a}}\nabla_{\mathbf{p}}{}^T\psi$ into $\det\left(\frac{1}{2\pi}\nabla\nabla^T\psi\right)$ at the largest order by $\frac{1}{N}$. Hence (S34) takes the form:

$$E(d,m,\tau) \cong \psi(\bar{\mathbf{a}},\bar{\mathbf{p}}) + \frac{1}{2}\log\det\left(\frac{1}{2\pi}\boldsymbol{\nabla}_{\mathbf{p}}\boldsymbol{\nabla}_{\mathbf{p}}{}^T\psi\right) + \frac{1}{2}\log\det\left(\frac{1}{2\pi}\boldsymbol{\nabla}_{\mathbf{a}}\boldsymbol{\nabla}_{\mathbf{a}}{}^T\psi\right). \tag{S35}$$

Second, due to the properties of the parameterizing function (S8) and the prior distribution of (S31) , $\det\left(\frac{1}{2\pi}\boldsymbol{\nabla}_{\mathbf{a}}\boldsymbol{\nabla}_{\mathbf{a}}{}^T\psi\right)$ at the minimum $(\bar{\mathbf{a}},\bar{\mathbf{p}})$ can be separated into d determinants corresponding to d PCs (again, at the largest order by $\frac{1}{N}$). Then from (S35) we come to the final expression:

$$E(d,m,\tau) \cong \psi(\bar{\mathbf{a}},\bar{\mathbf{p}}) + \frac{1}{2}\log\det\left(\frac{1}{2\pi}\boldsymbol{\nabla}_{\mathbf{p}}\boldsymbol{\nabla}_{\mathbf{p}}{}^T\psi\right) + \frac{1}{2}\sum_{k=1}^{d}\log\det\left(\frac{1}{2\pi}\boldsymbol{\nabla}_{\boldsymbol{\alpha}_k}\boldsymbol{\nabla}_{\boldsymbol{\alpha}_k}{}^T\psi\right). \tag{S36}$$

Here $\boldsymbol{\alpha_k} = \left(\alpha_k^1,\ldots,\alpha_k^m\right)$ is a vector with elements from (S9).

The second term in (S36) is a determinant of tridiagonal matrix (because of tridiagonality of $\mathbf{B}$ in (S26)). Thus, all components of (S36) can be computed quite fast, and the estimation time is limited mainly by the time of minimization (S19) for each triplet.

2. Simple example of NDM extraction

Data description Let us demonstrate a single NDM extraction step on a simple two-dimensional model dataset (see Figs. S6a, S6c) produced by a generator like (S6):

$$\mathbf{X}^n = \boldsymbol{\phi}(q^n) + \sigma_{\boldsymbol{\phi}}\cdot\zeta^n \tag{S37}$$

Here $\zeta^n \in \mathbb{R}^2$ is a Gaussian delta-correlated random process with noise intensity $\sigma_{\boldsymbol{\phi}} = 0.4$, and $\boldsymbol{\phi}$ is the following vector function:

$$\boldsymbol{\phi}(x) = \begin{pmatrix} 0.12x^3 + 0.89x^2 - 0.35x - 0.83 \\ 0.81x^3 - 0.26x^2 - 2.30x + 0.49 \end{pmatrix} \tag{S38}$$

which describes a nonlinear curve in $\mathbb{R}^2$. This curve is shown in Fig. S7a.

To produce scalar time series $q^1,\ldots,q^N$ we use time series of the variable y of the Lorenz system[10] in the chaotic regime corresponding to the classic Lorenz attractor:

$$\begin{aligned} \dot{x} &= 10(y-x) \\ \dot{y} &= 28x - y - xz \\ \dot{z} &= -\tfrac{8}{3}z + xy \end{aligned} \tag{S39}$$

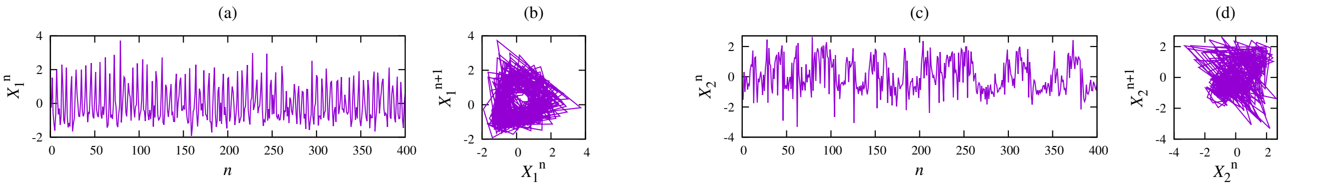

Figure S6. Two-dimensional dataset generated by (S37): time series of the observed variables (a, c) and their representation in time delayed coordinates (b, d).

In this regime the system's trajectory jumps between two symmetric wings of the attractor, while on each wing the trajectory rotates about the center of the wing. The average jumping period is 2.26 times more than the average rotation period.

We create the time series y^n by taking $N = 400$ values of the variable $y(t)$ with the time step $\Delta t = 0.14$ units and after the system has come to the attractor. This corresponds in average to 5.3 observations per one rotation and 33.2 jumps during the observation time interval, i. e. the time series y^n are smooth and cover the attractor. Finally, the time series q^n are produced by normalizing y^n to the standard deviation equal to 1. The time series q^n are shown in Fig. S8a. Also a time delayed representation of these time series is drawn in Fig. S8b to show the Lorenz attractor in a 2-D projection of the delayed coordinates phase space.

The observed noisy dataset $\mathbf{X}^n$ is shown on a 2-D plane of the variables (X_1, X_2) in Fig. S7b. The standard deviation of both $\phi_1(q^n)$ and $\phi_2(q^n)$ is approximately 1, so the noise level in the observed variables can be evaluated as $\sigma_{\boldsymbol{\phi}}$ and equals to 40%.

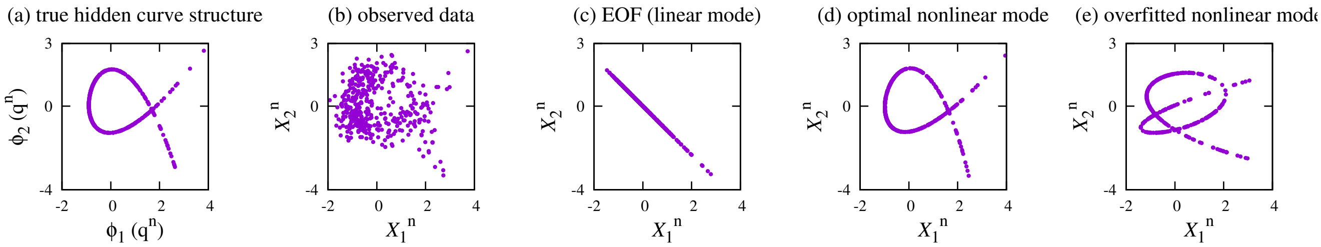

Figure S7. Representations in the space of the observed variables (X_1, X_2): (a) core nonlinear function (S38) of the system (S37); (b) observed time series of the system (S37); (c) too simple (linear) mode with hyperparameters $d = 2,\ m = 1,\ \tau = 10$; (d) nonlinear mode with optimal hyperparameters $d = 2,\ m = 3,\ \tau = 7.5$; (e) overfitted nonlinear mode with hyperparameters $d = 2,\ m = 7,\ \tau = 2.6$.

Results It is evident from Figs. S6b, S6d that the structure of the Lorenz attractor in the observed variables (X_1, X_2) is completely destroyed by the nonlinearity and noise. In this situation the NDM provides an answer to the important question: what is the optimal one-dimensional phase variable capturing the dynamical properties of the system? In our model example we know that this variable is given by projection of the data on the true curve in Fig. S7a.

The result of NDM extraction using our method can be seen in Figs. S7, S8. The nonlinear curve given by the parameters $\mathbf{a}$ of the NDM at optimal hyperparameters minimizing (S36) is shown in Fig. S7d. Obviously, this curve is very close to the true one, and the time series $\mathbf{p}$ of the hidden variable (Fig. S8e) demonstrate the structure of the Lorenz attractor in a delayed representation (Fig. S8f). In accordance with the optimality criterion (S33) the curves obtained at non-optimal hyperparameters are not similar to the true one: the too simple linear model of a mode (Fig. S7c) is not flexible enough, while the too complex model (Fig. S7e) gives complex overfitted curve capturing the particular noise realization in (S37). Neither linear (Fig. S8c,d), nor overfitted (Fig. S8g,h) modes can provide a good structure of the attractor in the hidden variables as the optimal one does.

3. Bayesian confidence interval estimation

In order to calculate Bayesian confidence intervals presented in the main text for NDMs of SST data, we first sampled the parameters $(\mathbf{a}, \mathbf{p})$ distributed by the approximate posterior distribution proportional to the integrand in (S32). The sampling was performed under the same approximation of the integrand in the vicinity of its maximum which holds in obtaining (S36) from (S32), i. e. we created Gaussian samples with mean $(\bar{\mathbf{a}}, \bar{\mathbf{p}})$ and the covariance matrix divided into blocks $\nabla_{\mathbf{p}} \nabla_{\mathbf{p}}{}^T \psi$ and $\nabla_{\boldsymbol{\alpha}_1} \nabla_{\boldsymbol{\alpha}_1}{}^T \psi, \ldots,\ \nabla_{\boldsymbol{\alpha}_d} \nabla_{\boldsymbol{\alpha}_d}{}^T \psi$ making $\mathbf{p}$ and $\boldsymbol{\alpha}_1, \ldots, \boldsymbol{\alpha}_d$ approximately independent Gaussian variables.

Then for each time moment n we cut 2.5% of the samples with the largest values of p^n and 2.5% of the samples with smallest values of p^n to obtain the 95% confidence interval for p^n.

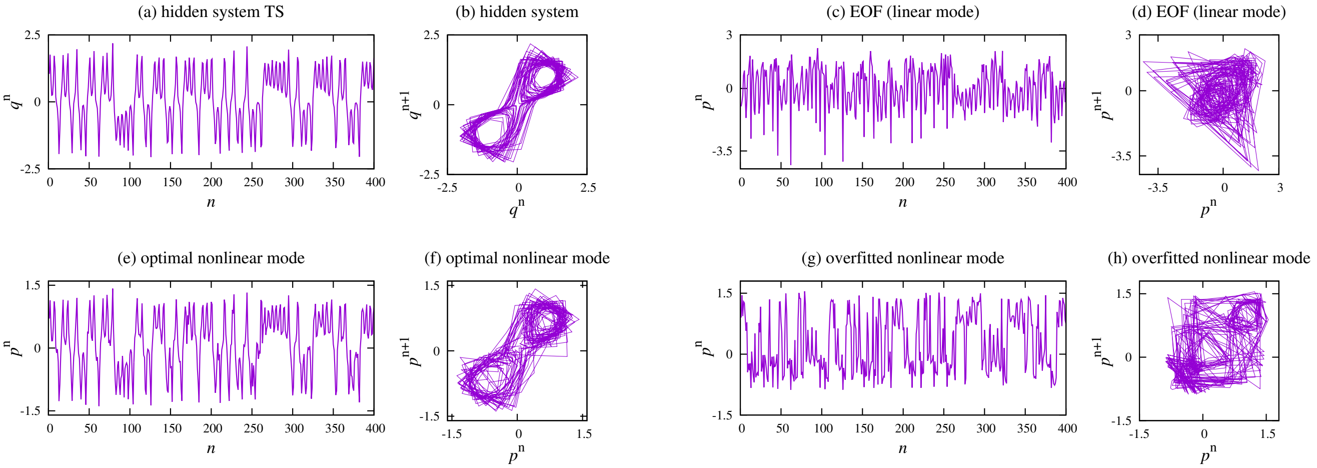

Figure S8. Time series of the hidden variable (a, c, e, g) and their representation in time delayed coordinates (b, d, f, h) for the following cases: (a, b) the system (S37); (c, d) too simple (linear) mode with hyperparameters $d = 2,\ m = 1,\ \tau = 10$; (e, f) nonlinear mode with optimal hyperparameters $d = 2,\ m = 3,\ \tau = 7.5$; (g, h) overfitted nonlinear mode with hyperparameters $d = 2,\ m = 7,\ \tau = 2.6$.

To calculate confidence intervals for NDM-based indices, we produced time series of these indices from each $(\mathbf{a}, \mathbf{p})$, i. e. created samples of the indices, and then estimated the 95% confidence interval for each time moment for each index in the same way as for $\mathbf{p}$ time series.

The Bayesian confidence intervals obtained by the way described above show the uncertainty in determining NDMs induced by the uncertainty of learning $\mathbf{a}$ and $\mathbf{p}$ for each NDM.

Generally, the confidence zone is more shallow for the hidden time series $\mathbf{p}$ than for the indices, because the uncertainties of the latter are influenced additionally by the uncertainty of $\mathbf{a}$.

References

1. Jolliffe, I. *Principal Component Analysis* (Springer-Verlag, 1986), 2 edn.
2. Jeffreys, H. *Theory of probability* (3rd ed. Oxford Univ. Press., 1961).
3. MacKay, D. *Information Theory, Inference, and Learning Algorithms* (Cambridge Univ. Press., 2003).
4. Barron, A., Rissanen, J. & Yu, B. The minimum description length principle in coding and modeling. *IEEE Transactions on Information Theory* **44**, 2743–2760 (1998).
5. Molkov, Y. I., Mukhin, D. N., Loskutov, E. M., Feigin, A. M. & Fidelin, G. A. Using the minimum description length principle for global reconstruction of dynamic systems from noisy time series. *Phys. Rev. E* **80**, 046207 (2009).
6. Erdelyi, A. *Asymptotic Expansions* (Dover, 1956).
7. Gelfand, A. E. & Smith, A. F. M. Sampling-based approaches to calculating marginal densities. *Journal of the American Statistical Association* **85**, 398–409 (1990).
8. Dempster, A., Laird, N. & Rubin, D. Maximum likelihood from incomplete data via the em algorithm. *Journal of the Royal Statistical Society, Series B* **39**, 1–38 (1977).
9. Loskutov, E. M., Molkov, Y. I., Mukhin, D. N. & Feigin, A. M. Markov chain monte carlo method in bayesian reconstruction of dynamical systems from noisy chaotic time series. *Phys. Rev. E* **77**, 066214 (2008). URL http://link.aps.org/doi/10.1103/PhysRevE.77.066214.
10. Lorenz, E. N. Deterministic Nonperiodic Flow. *J. Atmos. Sci.* **20**, 130–141 (1963). URL http://dx.doi.org/10.1175/1520-0469(1963)020%3C0130:dnf%3E2.0.co;2.
