# Supplementary figures and images for: Principal nonlinear dynamical modes of climate variability
Source: Sci Rep. 2015 Oct 22;5:15510. doi: 10.1038/srep15510 (PMC5155699; doi:10.1038/srep15510)

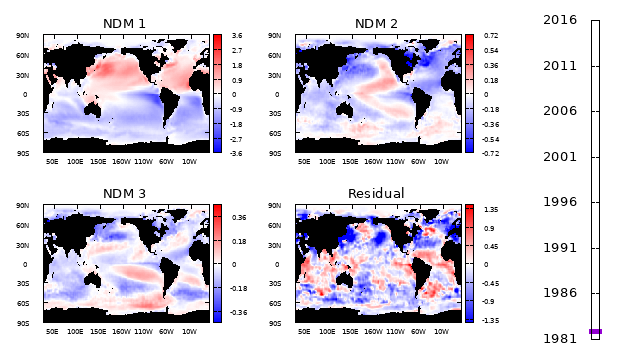

Supplement: Supplementary Animation [file srep15510-s2.gif]
